# Archetypal-Imaging and Mirror-Gazing

**DOI:** 10.3390/bs4010001

**Published:** 2013-12-24

**Authors:** Giovanni B. Caputo

**Affiliations:** DIPSUM, University of Urbino, via Saffi 15, 61029 Urbino, Italy; E-Mail: giovanni.caputo@uniurb.it

**Keywords:** archetype, *coniunctio oppositorum*, dissociation, empathy, hallucination, magic, *nigredo*, Pompei, synchronicity

## Abstract

Mirrors have been studied by cognitive psychology in order to understand self-recognition, self-identity, and self-consciousness. Moreover, the relevance of mirrors in spirituality, magic and arts may also suggest that mirrors can be symbols of unconscious contents. Carl G. Jung investigated mirrors in relation to the unconscious, particularly in *Psychology and Alchemy*. However, the relationship between the conscious behavior in front of a mirror and the unconscious meaning of mirrors has not been clarified. Recently, empirical research found that gazing at one’s own face in the mirror for a few minutes, at a low illumination level, produces the perception of bodily dysmorphic illusions of strange-faces. Healthy observers usually describe huge distortions of their own faces, monstrous beings, prototypical faces, faces of relatives and deceased, and faces of animals. In the psychiatric population, some schizophrenics show a dramatic increase of strange-face illusions. They can also describe the perception of multiple-others that fill the mirror surface surrounding their strange-face. Schizophrenics are usually convinced that strange-face illusions are truly real and identify themselves with strange-face illusions, diversely from healthy individuals who never identify with them. On the contrary, most patients with major depression do not perceive strange-face illusions, or they perceive very faint changes of their immobile faces in the mirror, like death statues. Strange-face illusions may be the psychodynamic projection of the subject’s unconscious archetypal contents into the mirror image. Therefore, strange-face illusions might provide both an ecological setting and an experimental technique for “imaging of the unconscious”. Future researches have been proposed.

## 1. Mirrors in Psychology

Mirrors have been studied in cognitive psychology in relationship to self-recognition, self-identity and self-consciousness. The attainment of a developmental stage of basic self-recognition is commonly gauged through reactions to a mirror [1,2,3]. Mirrored-self recognition involves the connection between the representation of a visual image that is external to the subject and the representation of the subject’s self. This process most probably requires the binding of visual information (*i.e*., the subject’s mirrored image), somaesthetic, kinaesthetic, affective and motor representations into a global representation of the subject’s self.

Mirrors are, by definition, virtually perfect “imitators” of the observer’s own bodily face, since mirror feedback is instantaneous in time. Moreover, if the mirror is flat and without visible imperfections, the reflected image is completely coherent in space with respect to the original visual stimulus. In addition to these perceptual and spatial characteristics, mirrors are perfect “imitators” of facial emotions and expressions, since they presumably produce unconscious mimicry and emotional contagion [4] within the subject itself by gazing at its own reflected image. In turn, unconscious mimicry can presumably produce empathic resonance [5] and emotional contagion within the subject. In other words, the mirror can create a sort of “closed loop” between perception, action and emotion within the observed/observing subject.

Contrary to a simplistic view that describes mirror gazing as equivalent to looking at static photos, some phenomenological investigations describe a more unsettling encounter with one’s mirrored double [6,7,8]. Merleau-Ponty [6,7] described the mirror as an object that allows both to perceive the subject’s own facial features and to apprehend its own body’s unity in a way which is different from that which is available from interoceptive, proprioceptive and exteroceptive sources. The subject becomes a spectator when it recognizes its mirrored image: seeing itself in the mirror is seeing itself as others see it. Therefore, mirror self-recognition exemplifies a troubled form of self-knowledge, since the mirror facilitates the subject’s alienation into its double. The decisive and unsettling impact of mirror self-recognition is the realization that the subject exists in an intersubjective space. This finding strongly distinguishes mirror self-recognition from self-identification in photos. The uncanny character of the mirrored image is due to intermingling of self and other representations within the subject–a process that is completely absent when identifying photos. “Thereupon I leave the reality of my lived me in order to refer myself constantly to the ideal, fictitious, or imaginary me, of which the specular image is the first outline. In this sense I am torn from myself, and the image in the mirror prepares me for another still more serious alienation, which will be the alienation by others.” ([7], p. 136).

Developmental, neurophysiological and neuropsychological studies showed that mirrored reflections are not equivalent to pictures and live videos [9]. Children show signs of self-recognition in photos much sooner than they are able to pass the mark test with mirrors [10]. On the other hand, children pass mirror versions of the mark task before the versions involving live videos [11]. The neural signatures for self-recognition differ depending upon whether using a mirror or a photo [12]. Some neuropsychological patients may not recognize themselves in mirrors (mirrored-self misidentification; [13]), while retaining their capacity to recognize themselves in photos [14]. Feinberg [15] proposed to group various syndromes that present alterations in the patient’s self-identity within the category of neuropathologies of the self. These produce an alteration in the regulation of the self-boundaries, either in the direction of the under-relatedness to personally significant aspects of the self (as mirrored-self misidentification) or in the direction of the over-relatedness to selected aspects of the world that the patient inappropriately over-incorporates into the self.

In connection to phenomenological experiences of alienation or dissociation by the subject in front of its reflected image [6,7,8], a relationship to out-of-body experiences [16] can be discussed. Experiments with virtual reality showed that a multi-sensory bodily self-representation is bound through the integration of visual virtual reality and touch information in experiments of spatial self-location [17,18,19]. During mirror self-recognition, a similar binding process is probably present for multi-sensory integration of visual (*i.e*., the mirrored image of the subject’s body), somatic, kinaesthetic, affective and motor representations into a global representation of the subject’s self.

**Figure 1:**
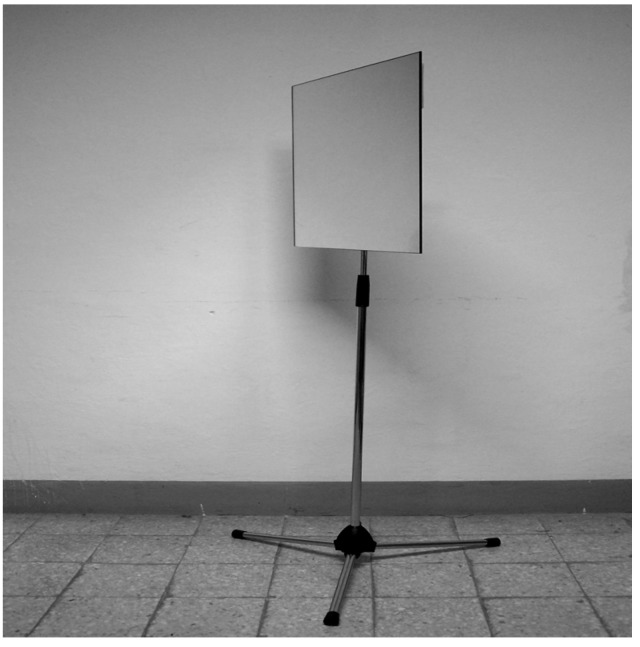
The mirror stand used in the experiment of mirror-gazing. The room should be without external light. A uniform illumination of the face (about 0.8 lux) can be obtained by placing a small lamp, or a candle, on the floor at some distance from the subject’s back [20,21]. The subject’s task is to stare at its eyes in the mirror.

Strange-face illusions in the mirror have been recently described during gazing at one’s own face reflected in the mirror for a few minutes at a low illumination level (Figure 1). Healthy observers sometimes see huge distortions of their own faces, but they often see monstrous beings, prototypical faces, faces of relatives and deceased, and faces of animals [20,21].

Strange-face illusions often involve the perception of strange-others who appears beyond the mirror, thus indicating the subject’s dissociation [21,22,23,24]. In general, naïve observers describe their feeling of losing control when strange-faces suddenly pop out from the mirror [21]. Dissociative experiences of strange-face illusions in healthy individuals typically dissipated after 15 min [23].

Consistent with these ideas of a dissociative process, observers wearing a full-face theatrical mask during mirror-gazing (e.g., a Japanese ko-omote mask of Noh theatre, [25]) have described how the facial features of the mask become animated (e.g., opening the physically closed mouth, as for lip movement or speaking, or shifting the animated eyes) and strange-faces in place of the physical mask are perceived.

In healthy individuals, strange-face illusions during mirror gazing usually involve the perception of one strange-face at a time. The duration of the illusion has been reported to be roughly seven seconds [21]. However, there are some healthy observers who describe intense flux or streaming experiences of continuously changing faces of unknown persons; the stream of new faces can last for a relatively long time. Only a few healthy individuals describe the perception of a second face, like another man/woman, just behind their face that instead becomes dark and unmoved like an inanimate mask. Alternatively, some schizophrenic patients describe the perception of multiple-others that fill the mirror surface surrounding the strange face [26]. Many patients were convinced that strange-face illusions were truly real and identify themselves with strange-face illusions, differently from healthy individuals who never identify with them [26]. This deficit in schizophrenia can be caused by pathological ego dysfunction [27].

**Figure 2:**
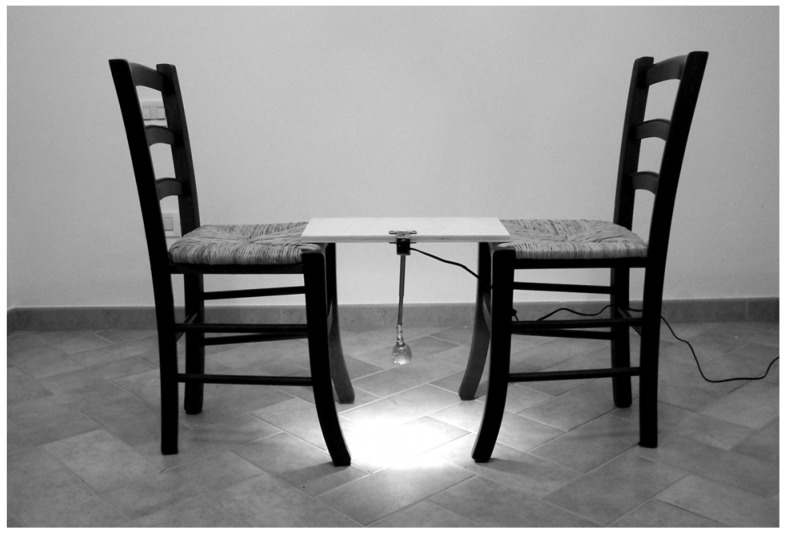
The interpersonal symmetric setting used for inter-subjective strange-face illusions [28]. The subject’s task is to stare into the eyes of the other participant.

Similar or even stronger strange-face illusions can be produced through an interpersonal setting (Figure 2) in which a pair of individuals are facing and gazing at each other in the eyes [28]. In such an inter-subjective setting, unconscious synchronization of responses is apparent in some dyads. On the basis of this finding, it is possible to hypothesize that strange-face illusions during mirror-gazing enact an interpersonal subject-other interaction in which the subject is facing its dissociative other located beyond or behind the mirror. A possible explanation of stronger strange-face illusions in some dyads with respect to mirror-gazing can be due to an increase of unconscious mimicry and emotional contagion within the dyad. Therefore, the symmetry of the interpersonal setting can lead to mirroring the bodily, affective, and psychological contents of strange-face illusions within these dyads.

Emotional responses to strange-face illusions are usually relatively intense in healthy individuals, and can be dramatic in some schizophrenic patients. Most frequent emotions are: surprise, interest and astonishment; other emotions include negative emotions such as moderate fear, anguish and fright, while positive emotions, such as hilarity and joyfulness, are rare.

As discussed above, self-recognition in mirrors is based on multiple cognitive processes. Therefore, strange-faces in the mirror are probably complex illusions involving different processes, from visual perception to motor facial mimicry, from self-other boundary to affective empathy, from unconscious contagion to conscious misidentification. Consequently, different mechanisms hypothesized as generative to strange-face illusions may be proposed. A first hypothesis is that strange-face illusions are perceptual and involve the Troxler effect [29]. This effect can explain merging of facial features into a uniform *silouette* of the facial contour; however, perception of entirely new faces remains unexplained. A second hypothesis is that prolonged adaptation to mirrored face disrupts multi-sensory binding between visual and bodily representations. This explanation can account for the frequent experiences of dissociation [23,24] and experiences that are similar to out-of-body perceptions of another person who is located beyond the mirror [21]. A third hypothesis is that low illumination can induce self-hypnosis and altered states of consciousness as a consequence of sensory deprivation [30], thus leading to perceptual and somatic pseudo-hallucinations of the subject’s face. A fourth hypothesis is that gazing at a low illumination can alter the self-other boundary [15] and by consequence can bring to mirrored-self misidentification and dissociation. A fifth hypothesis, which we prefer, can be based on emotions and empathy through facial mimicry and contagion [4,5], which can operate within the subject, resonating with its own face reflected in the mirror. Prototypical strange-faces could be a consequence of prototypical somatic/motor facial patterns of basic emotions. Self/other dissociation can facilitate “projection” of unconscious meaningful contents into external mirrored image. In the inter-subjective setting, some dyads can show unconscious synchronization of illusions as a consequence of synchronized facial mimicry between two individuals who are staring at each other in the eyes.

## 2. Strange-Face Illusions and Analytical Psychology

In relation to the concept of “projection”, the early idea of empathy, which was proposed by Lipps and Jung, is relevant. Lipps ([31], chapter 13) hypothesized that empathy is a form of objectification of the subject’s vital impulse, or activity into an external object that is different from the subject. Hence, the peculiar ability of empathy is that inanimate targets can become animated and appear alive. The targets that are animated by empathy appear as immediate *Dasein* and real, since the ego has become external and self-objective [31]. Jung ([32], chapter 7) hypothesized that Lipps’s idea of empathy is at the core of the psychodynamic concept of “projections” of the subject’s unconscious dissociative contents into others. Jung proposed an empathic personality trait which may be complemented by an opposite personality trait of abstraction in order to explain differences among individuals.

These ideas of empathy [31] and “projection” [32] can be relevant with respect to strange-face illusions. Strange-faces may be produced by unconscious contents that can only emerge within the subject’s consciousness when dissociated from the subject’s self and “projected” into the external mirrored-self image. In mirror-gazing, an overlapping between the conscious-self and the unconscious-self may provoke negative emotions and conflicts within the subject’s consciousness. Moreover, in inter-subjective gazing, a “projection” of the unconscious-self into another person can be much more easily produced and accepted by the subject’s consciousness.

Jung ([32], see Appendix 1) posited that the empathic personality trait is correlated to extroversion, while abstraction is correlated to introversion. If we assume that strange-face illusions are projections of unconscious contents, then Jung’s idea can make the prediction that extroverted subjects would show more proneness to strange-face illusions than introverted subjects. In agreement with this statement, we found that patients with major-depression [33] do not typically perceive strange-face illusions, or they perceive very faint changes of their immobile faces, like “death statues”, in the mirror [34]. In fact, according to Jung, depression is characterized by profound introjections of libido from the external world [35]. This may possibly reflect affective inhibition and blunting on the dissociative process which may require at least the potential for strong affective activation; a potential clearly dampened by depression [36]. Further support to Jung’s ideas comes from finding a positive correlation between individual differences in the proneness to strange-face illusions and empathic personality traits (“empathic-concern” and “fantasy” subscales of Interpersonal Reactivity Index [37]), as I have highlighted in recent research [38].

Jung ([32], see Definitions: Self) conceived the self as a totality of conscious and unconscious contents, together with a transcendent function that has the purpose to gain progressive awareness of unconscious contents. Another aspect of strange-face illusions concerns the integration of dissociated contents into the individual’s self. Integration starts through awareness of strange-faces, a process that is favoured by the fact that the mirror is also a physical object. Also, schizophrenic patients can take advantage of this awareness, since they can ground their (often dramatic) hallucinations elicited by mirror-gazing upon objectivity of the physical mirror. In this way, unconscious projections of dissociated contents can be integrated into the consciousness of the self. At present, only in a few cases have I had the opportunity to examine healthy naïve observers who underwent a number of mirror-gazing sessions over a number of days (about 5 to 20 sessions of 30–60 minutes). The strange-face that they perceived the first time in the mirror was by a person who was unknown or opposite to their conscious character and bodily appearance or sexual genre (e.g., a dark man, an old ancestor, a bad witch). The observers described their progressive comprehension of the identity of the strange-face during the sessions. This process of integration by awareness of the unknown aspect of the self can be similar to *imaginatio*, according to Jungian terminology [39].

Strange-face illusions could be classified according to archetypes described in analytical psychology [40]. In particular, the archetypes of shadow, anima/animus, mother, old-sage, hero, *puer*, and androgyne may be observed among strange-face illusions that are perceived by naïve observers [21] (see below). According to Jung, an archetype is structured into opposites. The process of individuation of the self presupposes the integration of opposites.

A phenomenological experience that is often described both by healthy and psychotic individuals is the “numinosity” of some strange-faces that they perceived. According to Jung, any time an unconscious archetypal content is constellated and emerges, it is characterized by numinosity, that is its fascinating power of attraction of the ego toward the unconscious, in a form of deep interest or even possession [41].

The synchronization of inter-subjective strange-face illusions [28] can be discussed in relation to Jung’s idea of synchronicity [42,43], which indicates the co-occurrence of events with roughly the same meaning at about the same time. According to Jung, synchronistic events arise whenever archetypes are constellated and, on the other side, synchronistic phenomena can be elicited by putting an individual into an unconscious state, as hypnosis or trance [41]. Archetypes of collective unconscious make synchronicity of individuals around numinous symbols. According to Jung, these events characterize telepathy between individuals and synchronistic events between the psychic and the physic. For example, numbers are archetypes of the order both of the physical world and of the self. Archetypal numbers not only express order of the world, but in addition the unconscious uses numbers as a factor that creates order [42].

Projection can probably be considered a form of synchronicity between the subject’s unconscious and the other’s body and mind. In the case of a pair of individuals, the synchronization of inter-subjective strange-face illusions [28] could be a form of Jung’s synchronicity [42] between the somatic, emotional, and psychic domains of the two individuals. This can produce a crossed projection of unconscious contents that are merged between the two individuals, with unconscious contents of one individual becoming also in part the unconscious contents of the other, on the basis of collective archetypes. According to Jung, this syzygy can create a crossed conjunction within the dyad ([39], chapter 5.6).

In addition to previous cognitive and analytical accounts of strange-faces, other aspects of these illusions need to be discussed in a wider cultural and anthropological context. In fact, unsettling experiences with mirrors is largely documented in arts and religion [44,45], magic, alchemy and spirituality [46]. In the following sections a review is made about these aspects of mirror usage, in order to gain a better understanding of strange-face illusions from these viewpoints. Then, we compare mirror illusions with Jung’s psychology of alchemy.

## 3. Mirrors in Magic and Alchemy

The magic power of mirrors dates back probably from the beginning of their invention. The art of using mirrors in divination and prediction of individual destiny (named *catoptromantia*) is found in the Dionysian testimonies [47]. The legend, chanted in the poem *Dionysiaca* by Nonnus of Panopolis, describes the killing of Dionysus (as a child) by his brothers the Titans, just when Dionysus gazes into the fascinating mirror (Figure 66b in [47] from *Birth of Dionysus*, Archaeological Museum of Bologna, Italy). A very large number of Greek and Roman vases show Bacchantes or Satyrs dancing in trance while gazing into a portable mirror. The great *Alexander mosaic* (named also *Battle of Isso*) at the Archaeological Museum of Napoli, Italy, shows an important detail in the lower centre part of the mosaic: a dying warrior gazes into the back side of his reflecting shield to see his ghost.

The most revealing information about the secret Dionysian mysteries is shown hermetically in the cycles of frescoes from the *Villa of Mysteries* in Pompei, Italy. An old Silenus offers some wine in a large reflecting silver bowl to a young man. The Silenus averts his gaze from the bowl, whereas the young man gazes into the mirror-reflecting bowl and has an astonished expression. Behind the young man there is another young man with identical facial features and clothes, but another expression of awareness—probably the same man that is doubled and yet has been initiated to the Mysteries. His double holds up a frightening mask which the drinking young man probably sees reflected in the bowl. Next to these characters sits a goddess, Ariadne or Aphrodite or mother Semele, with Dionysus who is euphoric or drunk, lying across her lap.

In the Renaissance, the use of magic mirrors is well documented. For example, John Dee, the Elizabethan magician, used a mirror made of oxydian stone (British Museum of London, England) to evoke angels and ghosts. The magical procedure is well illustrated by Rembrandt (*Faust and the magic mirror*; Figure 55 in [48]). The magic mirror is not used for reflecting faces or objects. It is placed at a distance of a few meters from the observer. Faust perceives the magical apparition out of the mirror in its left side (right outside the mirror with respect to Faust).

Paracelsus ([49], chapter 5) describes the construction of magic mirrors through the fusion of seven metals in order to establish a connection between macrocosm and microcosm. Jung ([39], chapter 3.4.D) quotes and discusses an intriguing text created by Paracelsus [50], in which the moon and the mirror are considered equals in their magic powers. According to Paracelsus, the mirror produces inter-relations between different human beings who have gazed or are gazing into it. Moreover, the moon is a mirror (see [39] chapter 3.4 for the complex implications of the moon in alchemy). Hence, the moon-mirror creates an explosive effect in the increase of the number of influences and contagions between human beings and astrological effects of the planets.

The Italian philosopher Julius Evola and co-workers (Ur group; [46]) describe the use of mirrors in order to perceive ethereal or spiritual presences. The same technique has been investigated in recent years with the so-called *psychomanteum* [51,52,53,54]. The setting requires a dark room, with the walls covered in black opaque curtain cloth, and the mirror is placed near the ceiling at a distance of a few meters from the observer. The mirror reflects the empty space above seated individuals who did not see their reflected body. This setting requires isolating the participant within the *psychomanteum* during a long session (60–90 minutes). Participants experience hallucinations of visual imagery, voices, sounds, light, body sensations and smell. Visual hallucinations of the mirrors are usually described as a halo around the mirror, or emitting light from the mirror, or colour changes in the light shining from the mirror [46].

In summary, the mirror in itself may create altered states of consciousness and trance when the mirror is displayed at a low illumination or when the subject has assumed drugs or alcohol. However, traditional and modern studies on magic reported above have never described strange-face illusions [20,21] when a subject gazes at its own image reflected in the mirror.

## 4. Mirrors in Carl G. Jung’s *Psychology and Alchemy*

In *Psychology and Alchemy* [48], Jung studied extensively the symbols connected to mirrors in relationship to the analysis of Wolfgang Pauli’s dreams. Jung’s investigation of mirrors is initially based on Schopenhauer’s idea [55] that Intellect is like a mirror, which reflects the Will ([48] part 2, chapter 3, dream 11 and dream 12). At the first apparition in the Pauli’s dreams, the mirror is being broken. In some ways, this fracture can also symbolize that Schopenhauer’s division between Will and Representation has been surpassed by the discovery of the unconscious.

In the course of Pauli’s dreams, the simple idea of the intellect as a mirror needs to be amplified in its symbolic meanings. In fact, Pauli’s dreams concerning mirrors are related to the problem of symmetry ([48], part 2, chapter 3, dream 25). Through the latter, the mirror is directly connected to the relationship between the conscious and the unconscious ([48], part 2, chapter 3, dream 25) and acknowledging parapsychology ([48], dream 25, note 112). The problem of symmetry in relation to mirrors occupies a number of dreams up to the “Great Vision” ([48], dream 59). Finally, the problem of symmetry leads toward the idea of synchronicity ([48], part 3, chapter 3.3). It can be noted that, in quantum physics, the symmetry is a form of synchronicity or, as Pauli preferred, of “complementarity”.

The symbols of mirror and symmetry can also be found in alchemy (Figure 209 in [48]). Although Jung [48] did not dedicate a specific essay to symbolic meanings of mirrors, it seems that mirrors and symmetries were most relevant for the development of the theory of synchronicity [42,43,56]. The *Opus* (e.g. the *Lapis*) is the term, used in alchemy, to describe the transformation of mind and matter. Alchemists after Paracelsus used the term *Unus Mundus* in order to indicate the same transformation, which leads different levels of reality toward unity or microcosm. According to Jung, the terms *Opus*, *Unus Mundus*, and synchronicity indicate the same psychoid reality in which physic and psychic, somatic and mental, mind and matter, are non-dualistic [39]. In the non-dualism of the psychoid, events which apparently occur at different dualistic levels actually co-occur. “*Unus est lapis, una medicina, unum vas, unum regimen, unaque dispositio*”—is said in the *Rosarium Philosophorum* ([39], chapter 3.4.B, note 316).

## 5. Strange-Face Illusions and Archetypal Imagery

Mirror-gazing at a low illumination produces visual illusions that are specific to one’s own face [20,21]. It is possible to hypothesize that strange-face illusions found in a controlled setting may be similar to pseudo-hallucinations obtained by *catoptromantia*, as described in Roman mosaics and frescoes. However, strange-face illusions seem different from hallucinations obtained with magic mirrors, as described in books by John Dee, Paracelsus, and other authors of the Renaissance. Strange-face illusions are clearly different from multi-sensory hallucinations produced within the *psychomanteum* [46,51,52,53,54]. Strange-face illusions seem more specific to reflect the projection of archetypes.

Strange-face illusions may provide both an ecological setting and an experimental technique for “imaging of the unconscious”. In fact, archetypal contents often characterize strange-face illusions. Jung’s ideas about alchemy [48] and particularly Jung’s psychological explanations of the different stages of the *opus* [39], can easily be applied to the phenomenological descriptions that naïve observers spontaneously produce about their experiences of strange-face illusions. From the findings in previous works [20,21], naïve observers very often described the archetype of shadow ([48], part 1). In some cases, as reported above, there is evidence for dissociation between the archetype of shadow and the social mask of the person. The feeling of the observers in response to strange-shadows is not pleasant. The archetype of the shadow is perceived very frequently in strange-face illusions of monsters, witches, skulls or cadavers. This finding is akin to the proposal to interpret the archetype of shadow in terms of alchemical *nigredo* (Figures 34 and 115 in [48]). Also, at this stage the relatively frequent perception of strange-animals in the mirror can be connected (Figures 90 and 175 in [48]). A very frequent archetypal content of strange-face illusions is an old man/woman, usually in the form of grand-father/mother, sometimes with black skin. Usually, observers in response to the old man/woman feel his/her intense numinosity: observers often state that the old man/woman seems to want to communicate something important. The strange old man/woman can be considered an archetype of the old-sage (or alchemical *magister*; Figures 168 and 179 in [48]). Another archetype that is also described by naïve observers is the archetype of anima/animus (Figure 9 and illustrations of the *albedo* in [48]), in the form of a strange-face of the opposite sex to the observer, usually having a cheerful presence. The numinous child (Figure 121 in [48]), usually seen with shining eyes, is also found in strange-face illusions by relatively few naïve observers. In strange-face illusions, a relatively rare archetype is the androgyne (Figures 164 and 208 in [48]). Therefore, these preliminary findings may indicate that the frequency of different archetypes can be correlated to the difficulty in the achievement of the process of individuation, according to Jung’s ideas of the archetype of the Self [39,48]. A direction of future research is to compile a questionnaire made of a list of possible archetypes that persons perceive in their strange-face illusions.

In psychiatric patients, strange-face illusions are usually characterized by *nigredo*. Most schizophrenics [26] perceive skulls, suicidal doubles, and dangerous felines. Some schizophrenics describe angels and gods in their hallucinations, but, when they are placed in front of the mirror, they discover that angelical presences are, in fact, satanic strange-face illusions. Some schizophrenics, but none of the healthy individuals, experienced multiple concomitant apparitions of different people in the mirror surrounding their strange-faces. In contrast, most patients with major-depression (MD; [33]) do not perceive strange-face illusions, since they perceive their immobile faces without emotions, as death statues in the mirror [34]. Further research may be done in patients with body-dysmorphic-disorder (BDD; [57]) and *anorexia nervosa* (AN; [58]), who suffer from their bodily imaginary appearance that, in terms of their actual physical body, is strongly dissociated. Notwithstanding the important psychopathology of BDD and AN patients, relevant differences in perceptual processes with respect to healthy individuals have not been found in previous studies. A hypothesis is that BDD patients can be very vulnerable to strange-face illusions and project their unconscious self into archetypal images in the mirror: for example, AN patients might perceive strange-faces of fat archetypal shadows. In general, strange-face illusions might constitute a technique of psychotherapy, since patients directly perceive unconscious dissociated parts of their selves, which may henceforth be recast into the ego’s awareness and possibly reintegrated. Re-absorbing projections involves affective empathy towards dissociated images. In alchemical psychology [39,59], this transformation is characterized as the *dealbatio* of *sol niger* into the specular bath of silver [60,61].

From previous research in healthy individuals [20,21], another finding is that a participant can often perceive different strange-face illusions, which can be classified into opposite archetypal contents or pairs of archetypes. Examples of strange-face archetypal pairs may be: the young man/woman and the old man/woman, the shining child and the very old man/woman, the unknown man and the unknown woman, the animal and the witch, and so on. This finding may agree with Jung’s idea that the individuation process is produced through *coniunctio oppositorum* [39] or crossing conjunctions of archetypal opposites [60].

In conclusion, in my opinion mirror gazing at a low illumination level could be a tool for integration of unconscious contents, which are usually projected, toward individuation of the self.
